# Predictive Ability of Visit-to-Visit Variability of HbA1c Measurements for the Development of Diabetic Kidney Disease: A Retrospective Longitudinal Observational Study

**DOI:** 10.1155/2022/6934188

**Published:** 2022-01-22

**Authors:** Yunyi Yan, Nozomi Kondo, Kentaro Oniki, Hiroshi Watanabe, Tadashi Imafuku, Yuki Sakamoto, Takuro Shigaki, Akari Maruyama, Hitomi Nakazawa, Tetsuya Kaneko, Ayami Morita, Akira Yoshida, Hitoshi Maeda, Toru Maruyama, Hideaki Jinnouchi, Junji Saruwatari

**Affiliations:** ^1^Division of Pharmacology and Therapeutics, Graduate School of Pharmaceutical Sciences, Kumamoto University, Kumamoto, Japan; ^2^Department of Biopharmaceutics, Graduate School of Pharmaceutical Sciences, Kumamoto University, Kumamoto, Japan; ^3^Department of Molecular Pathophysiology, Institute of Advanced Medicine, Wakayama Medical University, Wakayama, Japan; ^4^Diabetes Care Center, Jinnouchi Hospital, Kumamoto, Japan

## Abstract

**Aims:**

This study is aimed at clarifying the relationship between visit-to-visit variability of glycated hemoglobin (HbA1c) and the risk of diabetic kidney disease (DKD) and to identifying the most useful index of visit-to-visit variability of HbA1c.

**Methods:**

This clinic-based retrospective longitudinal study included 699 Japanese type 2 diabetes mellitus patients. Visit-to-visit variability of HbA1c was calculated as the internal standard deviation of HbA1c (HbA1c-SD), the coefficient of variation of HbA1c (HbA1c-CV), the HbA1c change score (HbA1c-HVS), and the area under the HbA1c curve (HbA1c-AUC) with 3-year serial HbA1c measurement data, and the associations between these indices and the development/progression of DKD were examined.

**Results:**

Cox proportional hazards models showed that the HbA1c-SD and HbA1c-AUC were associated with the incidence of microalbuminuria, independently of the HbA1c level. These results were verified and replicated in propensity score (PS) matching and bootstrap analyses. Moreover, the HbA1c-SD and HbA1c-AUC were also associated with oxidized human serum albumin (HSA), an oxidative stress marker.

**Conclusions:**

Visit-to-visit variability of HbA1c was an independent risk factor of microalbuminuria in association with oxidative stress among type 2 diabetes mellitus patients. HbA1c-AUC, a novel index of HbA1c variability, may be a potent prognostic indicator in predicting the risk of microalbuminuria.

## 1. Introduction

Diabetic kidney disease (DKD) is a microvascular complication of diabetes and is considered to be a renal symptom caused by long-term exposure to hyperglycemia [1]. Patients with DKD are not only at increased risk of developing end-stage kidney disease but are also at increased risk of cardiovascular morbidity and mortality [1, 2]. Therefore, optimizing glycemic control is most important for preventing the development and progression of DKD [2, 3]. Glycated hemoglobin (HbA1c) is traditionally used as an average blood glucose measurement to monitor blood glucose control in the treatment of type 2 diabetes [2, 3]. This rationale is based on clinical and observational evidence that lowering HbA1c reduces the risk of micro- and macrovascular complications of diabetes [2, 3]. However, a decreased HbA1c value alone has been reported to be insufficient to ensure optimal clinical outcomes in patients with type 2 diabetes [4]. The Action to Control Cardiovascular Disease in Diabetes (ACCORD) trial showed that the intensive reduction of blood glucose concentration only delayed the onset of albuminuria and was not associated with a decreased risk of other measures of renal dysfunction [5]. Therefore, other approaches—in addition to lowering HbA1c—are required to prevent the onset and progression of DKD.

Visit-to-visit variability of HbA1c, another indicator of glycemic control, has recently received attention for its relationship to the incidence of diabetic complications [6–16], although the importance of visit-to-visit variability in HbA1c to the risk of DKD is still under debate because of inconclusive evidence [16]. Since visit-to-visit variability of HbA1c is related to various factors (i.e., HbA1c), analyses to investigate the effect of visit-to-visit variability in HbA1c on the risk of DKD need to be adjusted for confounding factors [16]. Meanwhile, in many previous studies, visit-to-visit variability of HbA1c has been measured by the internal standard deviation of HbA1c (HbA1c-SD) and/or the coefficient of variation of HbA1c (HbA1c-CV) [6–11, 13]. HbA1c-SD and HbA1c-CV are influenced by the number of measurements and measurement intervals; thus, accurate quantitative evaluation of visit-to-visit variability of HbA1c has not been achieved. Recently, the HbA1c change score (HbA1c-HVS), which is calculated by dividing the number of times HbA1c changed by >0.5% (5.5 mmol/mol) by the total number of HbA1c measurements has been used as an index of visit-to-visit variability of HbA1c [14, 15]. However, it is unclear which index of visit-to-visit variability of HbA1c is most useful for predicting the risk of DKD, and there may be other useful indices. In order to clarify the causal relationship between visit-to-visit variability of HbA1c and the risk of DKD, it is necessary to conduct a comparative study using various indices.

In addition to clarifying the impact of visit-to-visit variability of HbA1c on the risk of DKD, it is also necessary to elucidate the mechanisms underlying the association between visit-to-visit variability of HbA1c and the development/progression of DKD. Oxidative stress is considered to be a key pathogenic factor in the association between visit-to-visit variability of HbA1c and diabetic complications [8–10, 17, 18]; however, the details are not clear, especially in DKD. We have recently reported that high levels of oxidized human serum albumin (HSA), a marker of oxidative stress, predict the development and progression of DKD [19]. Therefore, examining the association between oxidized HSA and visit-to-visit variability of HbA1c may help elucidate mechanisms in the relationship between visit-to-visit variability of HbA1c and the development/progression of DKD.

In the present study, in addition to three indices that have been used in previous studies (i.e., HbA1c-SD, HbA1c-CV, and HbA1c-HVS), the area under the HbA1c curve above or below the individual mean HbA1c (HbA1c-AUC) was also used as an index of visit-to-visit variability of HbA1c. We then investigated which index expressing visit-to-visit variability of HbA1c is the most associated with the development/progression of DKD, while also considering potential confounders. Moreover, we further investigated whether oxidized HSA could affect the visit-to-visit variability of HbA1c.

## 2. Materials and Methods

### 2.1. Subjects

In this study, the records of 728 consecutive type 2 diabetes mellitus patients who visited the Jinnouchi Clinic, Diabetes Care Center in Kumamoto, Japan, between July 1999 and October 2019 were reviewed. Among them, patients whose HbA1c level was measured ≥5 times within a 3-year period following the start of observation, and whose renal function was followed for ≥1 year, were included. Consequently, 699 subjects (477 males and 222 females) were included in the retrospective longitudinal study. For the diagnosis of type 2 diabetes, since all subjects enrolled in this retrospective study were Japanese patients, we applied the criteria of the Japan Diabetes Society (JDS), which are optimized for the Japanese population [20]. The diagnostic criteria are based on values of plasma glucose, HbA1c, typical symptoms of chronic hyperglycemia, and/or a prior diagnosis of diabetes, and the diagnostic cut-off values of plasma glucose and HbA1c for diabetes were a fasting plasma glucose level ≥ 126 mg/dl (≥7.0 mmol/l), 2 h oral glucose tolerance test value ≥ 200 mg/dl (≥11.1 mmol/l), casual plasma glucose level ≥ 200 mg/dl (≥11.1 mmol/l), and HbA1c ≥ 6.5% [20].

### 2.2. Study Design

The retrospective longitudinal study regarding the association between visit-to-visit variability of HbA1c and DKD was conducted as shown in Supplementary Figure 1. Considering possible intense changes of HbA1c in patients who started hypoglycemic treatment in primary care or replaced hypoglycemic medicines in use after transfer from another hospital, clinical data from 6 months after the first visit was used. In a 3-year period following the start of observation, HbA1c data were used to calculate the indices of visit-to-visit variability of HbA1c. After the 3-year period for calculating visit-to-visit variability of HbA1c, survival analyses with the baseline at the end of the 3-year period were performed to investigate the associations of visit-to-visit variability of HbA1c with the incidence of microalbuminuria and progression of the estimated glomerular filtration rate (eGFR) categories according to Kidney Disease: Improving Global Outcomes (KDIGO) GFR categories [21]. The length of follow-up for survival analyses was up to 10 years (median, 9.9 years; 2002-2019). In the analysis regarding the association between oxidized HSA and visit-to-visit variability of HbA1c, 194 subjects who underwent one time blood collection and the measurement of HbA1c more than 5 times in a 2.5-year period after blood collection were included. In the analysis, we calculated the visit-to-visit variability of HbA1c using HbA1c measurement data obtained for 2.5 years after oxidized HSA measurement and examined the association between oxidized HSA and visit-to-visit variability of HbA1c. The study protocol was approved by the institutional ethics committee of the Faculty of Life Sciences, Kumamoto University (Approval No.169), and the study was performed in accordance with the Declaration of Helsinki.

### 2.3. Indices of Visit-to-Visit Variability of HbA1c

In this study, the SD, CV, AUC, and HVS of HbA1c were employed as the index of visit-to-visit variability of HbA1c, denoted as HbA1c-SD, HbA1c-CV, HbA1c-AUC, and HbA1c-HVS, respectively. The 4 indices of visit-to-visit variability of HbA1c were calculated for each subject. HbA1c-HVS was calculated as the number of times HbA1c changed by >0.5% (5.5 mmol/mol) divided by the total number of HbA1c measurements [15]. For example, if a patient had 5 successive measurements of HbA1c with values of 6.7%, 7.0%, 7.8%, 7.4%, and 8.0%, then the HbA1c-HVS was calculated as 40% (i.e., 2/5 × 100%) [15]. HbA1c-AUC was calculated as the area of under the HbA1c curve above or below the individual mean HbA1c as shown in Supplementary Figure 2. Quartiles for each index were calculated and represented by Q1, Q2, Q3, and Q4. The lower quartile was denoted as Q1 (low HbA1c variability), and the upper quartile was denoted as Q4 (high HbA1c variability). In this study, HbA1c was measured using an ADAMS A1c HA-8181 (Arkray Inc., Tokyo, Japan), a glycohemoglobin analyzer that uses ion-exchange high-performance liquid chromatography assay to measure HbA1c and that has been certified by the Diabetes Control and Complications Trial and the National Glycohemoglobin Standardization Program (NGSP) reference assay (http://www.ngsp.org/docs/methods.pdf).

### 2.4. Endpoint Definitions

The development of DKD was evaluated by two assessments: the incidence of microalbuminuria and eGFR stage progression according to the KDIGO GFR category [21]. Survival analyses regarding the incidence of microalbuminuria were conducted among 533 subjects with normoalbuminuria at baseline. Normoalbuminuria was defined as a urine albumin- (ACR-) to-creatinine (Cr) ratio (Alb/Cr) of <30 mg/gCr or a negative urine dipstick test result, while microalbuminuria was defined as an Alb/Cr ≥ 30 mg/gCr or a positive urine dipstick test result followed by a subsequent Alb/Cr ≥ 30 mg/gCr. Survival analyses regarding eGFR stage progression were performed among subjects stratified according to the eGFR stage at baseline. The eGFR stage was determined according to the KDIGO GFR categories, as follows [21]: G1: normal, eGFR ≥ 90 ml/min/1.73 m^2^; G2: mild chronic kidney disease (CKD), eGFR: 60-89 ml/min/1.73 m^2^; G3a: mild-moderate CKD, eGFR: 45-59 ml/min/1.73 m^2^; G3b: moderate-severe CKD, eGFR: 30-44 ml/min/1.73 m^2^; G4: severe CKD, eGFR: 15-30 ml/min/1.73 m^2^; G5: end-stage kidney disease, eGFR < 15 ml/min/1.73 m^2^. In this study, G3a and G3b were combined as G3, since few subjects in the two groups experienced progression. A ≥25% decrease in eGFR was defined as progression of the eGFR stage. For example, if a patient had a baseline eGFR of 92 ml/min/1.73 m^2^ and a follow-up eGFR value equal to or lower than 69 ml/min/1.73 m^2^, this patient would be considered to have experienced progression from G1 (i.e., (92 − 69)/92 × 100% = 25%). The eGFR was calculated using the Modification of Diet in Renal Disease (MDRD) equation: eGFR (ml/min/1.73 m^2^) = 0.808 × 175 × Cr^−1.154^ × Age^−0.203^ × 0.742 (if female) [22].

### 2.5. Clinical Information

The clinical information that was used was obtained from medical records. This included HbA1c values, sex, age, duration of diabetes, height, weight, body mass index (BMI), systolic blood pressure (SBP), diastolic blood pressure (DBP), low-density lipoprotein cholesterol (LDL-C), high-density lipoprotein cholesterol (HDL-C), triglycerides, Alb, Cr, Alb/Cr, and eGFR. Type 2 diabetes mellitus was diagnosed according to the criteria of the JDS [20].

### 2.6. Determination of the Degree of Oxidized HSA

Among all subjects, we measured oxidized HSA in 194 subjects who underwent one time blood collection and the measurement of HbA1c > 5 times in a 2.5-year period after blood collection. HSA was collected by solid phase extraction and measured using time-of-flight mass spectrometry. The degree of oxidized HSA was calculated as follows: degree of oxidized HSA = [oxidized form of HSA/(oxidized form of HSA + unoxidized form of HSA)] × 100 [23].

### 2.7. Statistical Analysis

Continuous variables were compared by Student's *t*-test or a one-way ANOVA; categorical variables were compared by Fisher's exact test. The longitudinal associations of visit-to-visit variability of HbA1c with the incidence of microalbuminuria and eGFR stage progression were analyzed using Kaplan-Meier survival curves and Cox proportional hazards models. A comparison of the cumulative incidence between the groups was carried out using Kaplan-Meier survival curves with a log rank test. Multivariable adjusted hazard ratios (HRs) and 95% confidence intervals (CI) was calculated using a Cox proportional hazards model adjusted for age, sex, mean HbA1c over the 3-year period, duration of diabetes, SBP, LDL-C, eGFR, presence of ischemic heart disease and heart failure at baseline. Bootstrap analyses were performed to validate the associations of visit-to-visit variability of HbA1c with the incidence of microalbuminuria and eGFR stage progression [24]. One thousand replicated datasets were generated by random sampling with replacement [24]. Additionally, in order to reduce confounding bias, propensity score (PS) matching was performed to verify the results of the longitudinal analyses [24]. The PS was constructed using a logistic regression model. The following variables were included in the PS matching model: the mean HbA1c over the 3-year period, sex, age, HbA1c level, duration of diabetes, SBP, and BMI at baseline. Subjects were matched at a ratio of 1 : 1, using the nearest neighbor matching algorithm without replacement on the logit of the PS, with a caliper of width equal to 0.25 SD of the logit of the PS [24]. Besides, the longitudinal associations of continuous variables of the index of visit-to-visit variability of HbA1c with the incidence of microalbuminuria and eGFR stage progression were also analyzed using a Cox proportional hazards models adjusted for the same cofounders in the analysis containing category variables of variability of HbA1c. The association between oxidized HSA and visit-to-visit variability of HbA1c was analyzed using a multiple linear regression analysis with calculation of unstandardized partial regression coefficient (*B*), partial correlation coefficients (*β*), and standardized error (SE) adjusted by sex, mean HbA1c over the 2.5-year period, duration of diabetes, SBP, eGFR, and BMI. *p* values of <0.05 were considered to be statistically significant. PS matching was performed with a contributed R package (“Matching”) using the R software program (version 3.6.3, R Foundation for Statistical Computing, Vienna, Austria). Other statistical analyses were performed using the SPSS software package for Windows (Version 23.0, IBM Japan Ltd., Tokyo, Japan).

## 3. Results

A total of 699 type 2 diabetes mellitus patients (477 males and 222 females) were included in this study. Clinical characteristics of all subjects are shown in Table 1. At the start of the observation, the mean duration of diabetes was 9.0 ± 7.8 years, and the mean HbA1c was 7.7 ± 1.5%. The mean number of HbA1c measurements over the 3-year period was 28 ± 10 times (at least 5 measurements per subject), and the mean HbA1c over the 3-year period was 7.7 ± 1.1% (Table 1). The mean and quartile values of the 4 indices of visit-to-visit variability of HbA1c (HbA1c-SD, HbA1c-CV, HbA1c-AUC, and HbA1c-HVS) are shown in Supplementary Table 1. To observe the characteristics of subjects with low and high visit-to-visit variability of HbA1c, the overall subjects were divided by quartiles of the 4 indices, respectively, and results are shown in Supplementary Table 2-5. We found that whichever index was employed to measure the visit-to-visit variability of HbA1c, subjects with high HbA1c variability were characterized by significant younger age and higher mean HbA1c, BMI, SBP, triglycerides, and LDL-C values in comparison to those with low HbA1c variability (Supplementary Table 2-5).

**Figure 1:**
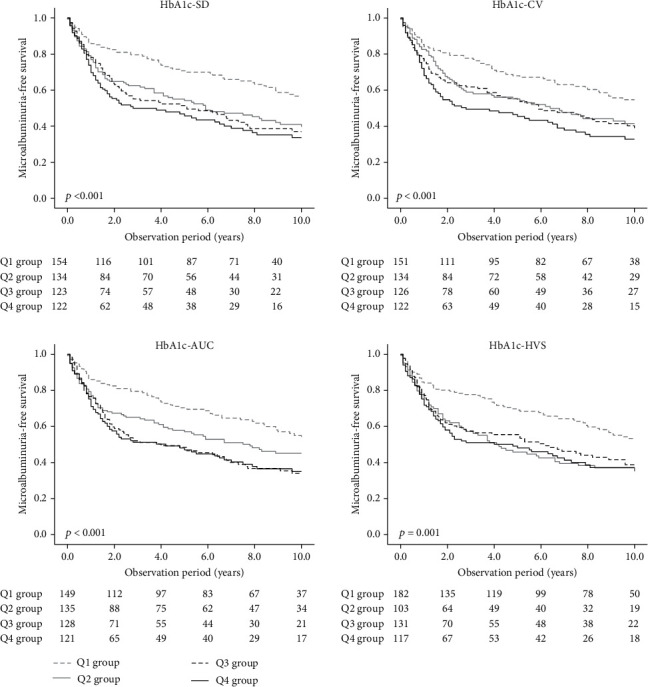
Kaplan-Meier curves for microalbuminuria-free survival among subjects divided by quartiles of the 4 indices of visit-to-visit variability of HbA1c. The quartiles of each index are represented by Q1, Q2, Q3, and Q4. The lower quartile is denoted as Q1, and the upper quartile is denoted as Q4. The comparison of the cumulative incidence among groups was carried out using a log rank test. HbA1c-SD: internal standard deviation of HbA1c; HbA1c-CV: coefficient of variation of HbA1c; HbA1c-AUC: area under the HbA1c curve; HbA1c-HVS: HbA1c change score.

Microalbuminuria is an early manifestation of the progression of DKD. In the present study, we performed survival analyses to investigate the association of visit-to-visit variability of HbA1c with the incidence of microalbuminuria among 533 subjects who had normoalbuminuria at the baseline of the survival analyses. The subjects were divided into 4 groups based on the quartiles of the 4 indices (HbA1c-SD, HbA1c-CV, HbA1c-AUC, and HbA1c-HVS). The incidence of microalbuminuria at endpoint was 52% (incidence rate: 10.8 cases per 1000 person-year). For all indices, Kaplan-Meier curves showed that the incidence of microalbuminuria differed among the 4 groups (Figure 1), and multivariable Cox proportional hazards models showed that the incidence of microalbuminuria in the Q4 groups of HbA1c-SD, HbA1c-CV, and HbA1c-AUC was higher in comparison to their respective Q1 groups (Table 2). Additionally, the risk was verified by a bootstrap analysis using 1,000 replicated datasets.

eGFR decline is another manifestation of the progression of DKD; thus, we also performed survival analyses to investigate the association of visit-to-visit variability of HbA1c with eGFR stage progression. First, we investigated the association of visit-to-visit variability of HbA1c with eGFR stage progression from stage G1 among 280 subjects who were in G1 at the baseline of the survival analyses. Subjects of stage G1 were divided into 4 groups based on the quartiles of the 4 indices (HbA1c-SD, HbA1c-CV, HbA1c-AUC, and HbA1c-HVS), respectively. The incidence of eGFR stage progression from stage G1 was 75% (incidence rate: 16.1 cases per 1000 person-year). Kaplan-Meier curves showed the incidence of eGFR stage progression from G1 among each group of the 4 indices (Supplementary Figure 3), and the multivariable Cox proportional hazards models showed that the eGFR stage progression from G1 was higher in the Q4 groups of HbA1c-SD and HbA1c-CV in comparison to their respective Q1 groups (Supplementary Table 6). The bootstrap analysis verified the associations of HbA1c-SD and HbA1c-CV with the incidence of eGFR stage progression from G1. Next, we investigated the association between visit-to-visit variability of HbA1c and eGFR stage progression from G2 or G3. However, there was no association between visit-to-visit variability of HbA1c and eGFR stage progression from G2 or from G3 (Supplementary Figure 4-5, Supplementary Table 7).

**Figure 2:**
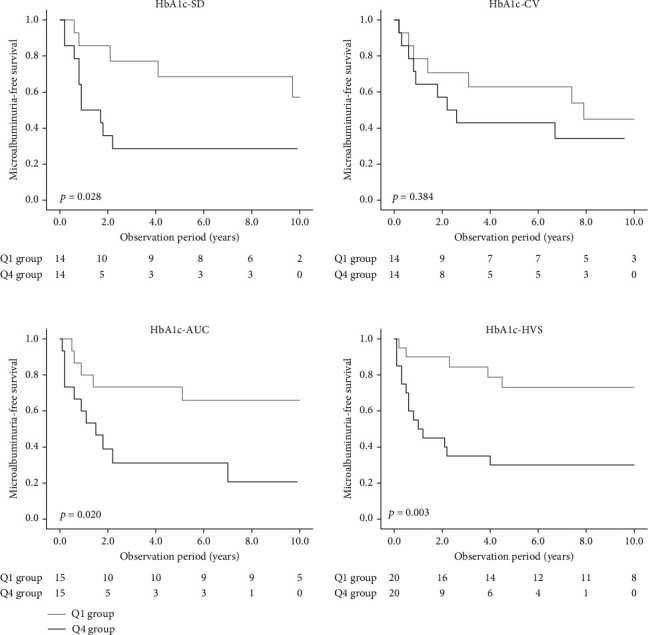
Kaplan-Meier curves for the microalbuminuria-free survival among PS-matched subjects from Q1 and Q4 group of each index of visit-to-visit variability of HbA1c. The lower quartile is denoted as Q1, and the upper quartile is denoted as Q4. The comparison of the cumulative incidence among groups was carried out using a log rank test. HbA1c-SD: internal standard deviation of HbA1c; HbA1c-CV: coefficient of variation of HbA1c; HbA1c-AUC: area under the HbA1c curve; HbA1c-HVS: HbA1c change score.

Subjects with high visit-to-visit variability of HbA1c were characterized by a higher HbA1c level, younger age, and undesirable clinical parameters, such as higher SBP (Supplementary Table 2-5), which may affect the development of DKD. Considering the impact of confounding bias on the result, we performed PS-matching among subjects in the Q1 (the lowest variability group) and Q4 (the highest variability group) groups of each index to further confirm the association of visit-to-visit variability of HbA1c with the incidence of microalbuminuria as well as eGFR stage progression from G1. The information of the PS-matched subjects can be found in Supplementary Table 8-9, which showed there was no significant difference in the variables used in PS matching between matched subjects. Survival analyses were conducted to compare the risk of microalbuminuria and eGFR stage progression from G1 between the matched subjects with the highest and lowest visit-to-visit variability of HbA1c. In the analyses for HbA1c-SD, HbA1c-AUC, and HbA1c-HVS, the incidence of microalbuminuria was higher in the subjects with the highest HbA1c variability in comparison to those with the lowest HbA1c variability (Figure 2, Table 2). However, regarding eGFR stage progression, no significant difference was found in the risk of eGFR stage progression from G1 between the highest and lowest HbA1c variability groups; this was found with all indices (Supplementary Figure 6, Supplementary Table 6).

Besides of analyzing the associations between category variables of variability of HbA1c, which were represented by the quartile groups, with incidence of microalbuminuria and eGFR stage progression in the Cox proportional hazards models, we also analyzed the continuous variables of variability of HbA1c in the Cox proportional hazards models. Increases in HbA1c-SD, HbA1c-CV, and HbA1c-AUC values were associated with an increase in the risk of microalbuminuria onset as well as the eGFR stage progression from G1 (Table 3).

Additionally, we investigated the association between visit-to-visit variability of HbA1c and oxidative stress. The degree of oxidized HSA was determined in 194 subjects, and the subjects were divided into 4 groups based on the quartiles of HbA1c-AUC and HbA1c-SD over a 2.5-year period. Multiple linear regression models showed significant associations between HbA1c-AUC and HbA1c-SD with the degree of oxidized HSA, and oxidized HSA was higher in the subjects with the highest HbA1c variability in comparison to those with the lowest HbA1c variability (Table 4).

## 4. Discussion

The present longitudinal study showed that the visit-to-visit variability of HbA1c was associated with the incidence of microalbuminuria in type 2 diabetes mellitus patients, independently of HbA1c. In addition, this is the first study to show that the HbA1c-AUC was associated with the incidence of microalbuminuria among the indices of visit-to-visit variability of HbA1c. Furthermore, oxidized HSA was associated with the HbA1c-AUC and HbA1c-SD, independently of the mean HbA1c. These results emphasize the importance of visit-to-visit variability of HbA1c in the early stage of DKD in relation to oxidative stress and indicate the potential of HbA1c-AUC as a novel predictive marker of the incidence of microalbuminuria.

A novel feature of our study was our use of four indices (HbA1c-SD, HbA1c-CV, HbA1c-AUC, and HbA1c-HVS) of visit-to-visit variability of HbA1c and the comparison of their predictive ability for the development of DKD. Dozens of studies have reported the association between HbA1c-SD and/or HbA1c-CV and the appearance of albuminuria and/or eGFR decline [8–11, 13]; however, studies on HbA1c-AUC and HbA1c-HVS are rare [14, 25]. Our study reveals that HbA1c-AUC quartiles were significantly associated with the appearance of microalbuminuria, the same as HbA1c-SD. Results from the bootstrap analysis and PS-matching analysis supported this association. Of note, the HRs of the quartiles of visit-to-visit variability of HbA1c for microalbuminuria showed an increasing trend from the lower quartile to the upper quartile in the results of HbA1c-AUC, which was not observed in the results of the other indices (Table 2). The two conventional indices (i.e., HbA1c-SD and HbA1c-CV) are calculated with a discrete HbA1c value and are thought to be affected by the number of HbA1c measurements and the measurement intervals. In contrast, HbA1c-AUC, is calculated nearly as the definite integral of a curve that describes the variation of HbA1c, which takes the continuous change between two discrete HbA1c value into account, thus considered to be less influenced by the frequency of measurement. This may be why the tendency for the incidence of microalbuminuria to increase from the lower quartile to the upper quartile was only observed for HbA1c-AUC in the present study. Taken together, HbA1c-AUC may be a candidate predictor of microalbuminuria risk.

The mechanisms underlying the association between visit-to-visit variability of HbA1c and diabetic complications are not fully understood; however, oxidative stress and subsequent endothelial dysfunction due to the overproduction of the superoxide radical by glycemic variability are commonly considered to be key pathogenic factors [8–10, 17, 18, 26]. Conversely, oxidative stress may also affect HbA1c variability because it is associated with impaired insulin secretion from beta cells and impaired glucose uptake and utilization by hepatocytes and skeletal muscle cells [27]. Some oxidative stress markers, such as 8-iso-prostaglandin F2*α*, thiobarbituric acid-reactive substance, and 8-hydroxydeoxyguanosine were reported to be positively correlated with visit-to-visit variability of HbA1c [28]. In this study, we found that the degree of oxidized HSA, a marker of oxidative stress [19, 23, 29], affects HbA1c-AUC and HbA1c-SD, independently of the mean HbA1c value. We recently reported that oxidized HSA could be an early predictive marker of a declining renal function in patients with type 2 diabetes [19]. Therefore, visit-to-visit variability of HbA1c may influence the decline in the renal function of patients with type 2 diabetes in close association with the oxidative stress status. Moreover, since multiple measurements of HbA1c (i.e., multiple visits to the hospital and blood sampling) are required to calculate the visit-to-visit variability of HbA1c, we suggest that the single measurement of oxidized HSA, although not a completely predictive marker, may be useful for predicting subsequent visit-to-visit variability of HbA1c.

This study showed that the visit-to-visit variability of HbA1c is an independent risk factor for microalbuminuria among type 2 diabetes patients, emphasizing the importance of optimizing an unfluctuating HbA1c to prevent early development of DKD. Higher visit-to-visit variability of HbA1c may be the consequence of suboptimal diabetes management and less responsible behaviors, such as poor treatment adherence and unhealthy habits [30]. Preferred pharmacological strategies, such as new antidiabetic drugs combined with basal insulin or metformin as well as lifestyle modification, including exercise training (i.e., resistance exercise) and dietary intervention (i.e., low carbohydrate diet) may be potential strategies to alleviate glycemic variability [31–34]. Besides, seasonal changes contribute to HbA1c variability, with high and low HbA1c values generally observed in winter and summer, respectively [35]. In our study, we observed a similar influence of seasonal changes on the variation of HbA1c: the peak and trough HbA1c values appeared in March and August, respectively (Supplementary Figure 7). Yamada et al. reported that add-use of SGLT2 inhibitors attenuated the tendency for HbA1c to worsen towards the winter season [36], suggesting that a moderate adjustment in antiglycemic medication is a valuable strategy for treating seasonal variation in HbA1c.

HbA1c-HVS, weighted for clinically significant changes in HbA1c (>0.5%), was also measured in this study [15]. However, our data reveal that HbA1c-HVS was not associated with the development of DKD, which is likely caused by the distribution bias of the subjects; in our study, a high percentage of subjects (31%) had an HbA1c-HVS value of 0% and was classified into the Q1 group. Li et al. reported the role of HbA1c-HVS in the prediction of cardiovascular events and several microvascular complications among newly diagnosed type 2 diabetes patients [25], while HbA1c-HVS has not been widely used, the utility of HbA1c-HVS in representing visit-to-visit variability of HbA1c and predicting diabetic complications requires further exploration.

In this study, subjects with high visit-to-visit variability of HbA1c were characterized by younger age, higher BMI, blood pressure and triglyceride values, and lower HDL-C. Part of these characteristics were also observed in people at risk of developing CVD [37], which was reported to be associated with visit-to-visit variability in previous studies [8, 38–42]. Thus, subjects with high visit-to-visit variability of HbA1c may have a higher risk of developing CVD, along with clinical characteristics of CVD; however, whether there are biological mechanisms underlying the association between the clinical characteristics of CVD and high visit-to-visit variability of HbA1c is unclear.

The present study was associated with some limitations. In the present study, the effect of the HbA1c level on the association between visit-to-visit variability of HbA1c and DKD was minimized by adjusting for the mean HbA1c during the 3-year period. However, prescription patterns, such as intensive treatment, monotherapy or polytherapy, which can affect HbA1c levels, were not accounted for in the multivariable analysis, and this should be further investigated in a future study. The incidence of microalbuminuria in the subjects of this study was relatively high at 52% [43]. Some of the study subjects had been transferred from other hospitals and were under special management for diabetes at the diabetes center; these patients had a history of severe diabetes or suboptimal management, which may have contributed to the high incidence of microalbuminuria. In addition, the presence of an older age (48% of the subjects were over 60 years old), longer duration of diabetes (50% of the subjects had diabetes for more than 10 years), and more comorbidities such as hypertension (30%), dyslipidemia (54%), and macrovascular disease (25%) at baseline may also have been associated with the high incidence of microalbuminuria in this study. This study had a relatively small sample size, and subjects were limited to a Japanese population; accordingly, the results should be interpreted with some caution. Further investigations in a large-scale population involving subjects of different ethnicity or in different countries are necessary to confirm the findings.

The abovementioned limitations may partly explain why we did not find a significant association between visit-to-visit variability of HbA1c and eGFR stage progression. The effects of visit-to-visit variability of HbA1c were likely to have been masked by unadjusted confounding factors. Also, the small sample size in the analysis of eGFR stage progression may have been insufficient to reach statistical significance. On the other hand, another explanation is that the appearance of albuminuria and GFR decline occur independently of each other as risk factors in patients with diabetes [44]. Although visit-to-visit variability of HbA1c is associated with the appearance of microalbuminuria, it may not be associated with early eGFR stage progression, and two previous studies reported similar findings [9, 45].

The present study was also associated with some strengths. In addition to HbA1c-SD, HbA1c-CV and HbA1c-HVS and a new index, HbA1c-AUC, were employed and explored in this study. Since the subjects in our study had received monthly glycemic checkups in a diabetes clinic for a number of years, a large number of serial HbA1c measurements for individual patients were available in the analyses, which ensured the reliability of our findings. Additionally, a bootstrap analysis and sensitivity analysis were conducted to verify the robustness of the findings. Furthermore, we showed that oxidized HSA was associated with subsequent visit-to-visit variability of HbA1c.

## 5. Conclusions

In conclusion, our study showed that visit-to-visit variability of HbA1c is a novel predictor of microalbuminuria, independent of HbA1c. In particular, HbA1c-AUC could be an accurate and stable indicator of visit-to-visit variability of HbA1c. Moreover, we suggest that oxidized HSA may be a predictive marker of the visit-to-visit variability of HbA1c. This study provided evidence to support that visit-to-visit variability of HbA1c plays an important role in the development of DKD among type 2 diabetes patients and pointed out the importance of paying attention to the control of HbA1c variability in diabetes treatment.

## Figures and Tables

**Table 1:** Clinical characteristics of all subjects.

	All subjects (*n* = 699)
Male/female	477/222
Start of the observation	
Age (years)	56.1 ± 10.4
Duration of diabetes (years)	9.0 ± 7.8
HbA1c (%)	7.7 ± 1.5
BMI (kg/m^2^)	24.3 ± 4.0
SBP (mmHg)	135.8 ± 18.1
DBP (mmHg)	82.1 ± 11.4
HDL-C (mmol/l)	1.4 ± 0.4
LDL-C (mmol/l)	3.2 ± 0.9
Triglycerides (mmol/l)	1.6 ± 1.4
3-year period following the start of observation	
Mean HbA1c (%)	7.7 ± 1.1
Number of HbA1c measurements (times)	28 ± 10

Data are shown as the number or the mean ± SD. BMI: body mass index; SBP: systolic blood pressure; DBP: diastolic blood pressure; HDL-C: high-density lipoprotein cholesterol; LDL-C: low-density lipoprotein cholesterol; SD: standard deviation.

**Table 2:** Association of the risk of microalbuminuria with visit-to-visit variability of HbA1c, measured as 4 indices using a Cox proportional hazards model.

Index	HR (95% CI)^a^	*p* value^b^	*p* value^c^	PS-matched subjects	
				HR (95% CI)	*p* value^b^
HbA1c-SD					
Q1	1	—	—	1	—
Q2	1.60 (1.11-2.30)	0.011	0.012		
Q3	1.52 (1.00-2.30)	0.050	0.076		
Q4	**1.77 (1.12-2.79)**	**0.014**	**0.015**	**3.18 (1.06-9.58)**	**0.040**
HbA1c-CV					
Q1	1	—	—	1	—
Q2	1.51 (1.06-2.17)	0.024	0.022		
Q3	1.19 (0.79-1.77)	0.404	0.407		
Q4	1.61 (1.06-2.44)	0.026	0.025	1.54 (0.57-4.17)	0.391
HbA1c-AUC					
Q1	1	—	—	1	—
Q2	1.28 (0.89-1.85)	0.183	0.158		
Q3	1.51 (1.01-2.26)	0.047	0.027		
Q4	**1.60 (1.02-2.49)**	**0.039**	**0.032**	**3.29 (1.13-9.61)**	**0.027**
HbA1c-HVS					
Q1	1	—	—	1	—
Q2	1.52 (1.07-2.17)	0.019	0.018		
Q3	1.19 (0.82-1.72)	0.365	0.371		
Q4	1.33 (0.83-2.12)	0.235	0.258	4.23 (1.51-11.85)	0.006

The quartiles of each index are represented by Q1, Q2, Q3, and Q4. The lower quartile is denoted as Q1, and the upper quartile is denoted as Q4. ^a^Adjusted by age, sex, mean HbA1c over the 3-year period, duration of diabetes, SBP, LDL-C, and presence of ischemic heart disease and heart failure at baseline. ^b^Analyzed using a Cox proportional hazards model. ^c^Analyzed using a bootstrap analysis based on 1,000 replicated datasets. HR: hazard ratio; CI: confidence interval; PS: propensity score; HbA1c-SD: internal standard deviation of HbA1c; HbA1c-CV: coefficient of variation of HbA1c; HbA1c-AUC: area under the HbA1c curve; HbA1c-HVS: HbA1c change score; SBP: systolic blood pressure; LDL-C: low-density lipoprotein cholesterol.

**Table 3:** Association of the risk of microalbuminuria and eGFR stage progression with continuous variables of visit-to-visit variability of HbA1c, measured as 4 indices using a Cox proportional hazards model.

Index	Incidence of microalbuminuria		eGFR stage progression from G1		eGFR stage progression from G2		eGFR stage progression from G3	
	HR (95% CI)^a^	*p* value	HR (95% CI)^a^	*p* value	HR (95% CI)^a^	*p* value	HR (95% CI)^a^	*p* value
HbA1c-SD	**1.18 (1.01-1.37)**	**0.033**	**1.23 (1.06-1.43)**	**0.008**	1.09 (0.86-1.37)	0.483	1.09 (0.73-1.61)	0.675
HbA1c-CV	**1.17 (1.03-1.34)**	**0.019**	**1.21 (1.05-1.39)**	**0.008**	1.09 (0.89-1.33)	0.400	1.09 (0.77-1.53)	0.634
HbA1c-AUC	**1.20 (1.03-1.40)**	**0.019**	**1.22 (1.05-1.42)**	**0.010**	1.09 (0.84-1.43)	0.509	1.04 (0.67-1.62)	0.848
HbA1c-HVS	1.00 (0.85-1.17)	0.952	1.03 (0.88-1.21)	0.735	1.18 (0.96-1.46)	0.105	0.99 (0.57-1.69)	0.958

^a^Adjusted by age, sex, mean HbA1c over the 3-year period, duration of diabetes, SBP, LDL-C, eGFR (in the analysis of eGFR stage progression), and presence of ischemic heart disease and heart failure at baseline. eGFR: estimated glomerular filtration rate; HR: hazard ratio; CI: confidence interval; HbA1c-SD: internal standard deviation of HbA1c; HbA1c-CV: coefficient of variation of HbA1c; HbA1c-AUC: area under the HbA1c curve; HbA1c-HVS: HbA1c change score; SBP: systolic blood pressure; LDL-C: low-density lipoprotein cholesterol.

**Table 4:** Association between degree of oxidized HSA and visit-to-visit variability of HbA1c, as assessed by a multiple linear regression model.

Index	*B* ^a^	*β*	SE	*p* value
HbA1c-SD				
Q1	—	—	—	—
Q2	1.75	0.15	0.91	0.056
Q3	1.84	0.15	0.97	0.059
Q4	**2.35**	**0.19**	**1.18**	**0.047**
HbA1c-AUC				
Q1	—		—	—
Q2	**1.87**	**0.16**	**0.92**	**0.044**
Q3	**2.03**	**0.17**	**0.99**	**0.041**
Q4	**2.56**	**0.21**	**1.16**	**0.029**

The quartiles of each index are represented by Q1, Q2, Q3, and Q4. The lower quartile is denoted as Q1, and the upper quartile is denoted as Q4. ^a^Adjusted by sex, mean HbA1c over the 2.5-year period, duration of diabetes, SBP, eGFR, and BMI. HSA: human serum albumin; HbA1c-SD: internal standard deviation of HbA1c; HbA1c-AUC: area under the HbA1c curve; B: unstandardized partial regression coefficient; *β*: partial correlation coefficients; SE: standardized error SBP: systolic blood pressure; eGFR: estimated glomerular filtration rate; BMI: body mass index.

## Data Availability

The datasets generated during and/or analyzed during the current study are available from the corresponding author on reasonable request.

## Supplementary Materials

Supplementary Materials Supplementary Figure 1: study design to investigate the association between visit-to-visit variability of HbA1c and DKD risk. Supplementary Figure 2: calculation method of HbA1c-AUC. Supplementary Figure 3: Kaplan-Meier curves for eGFR stage progression-free survival from G1 among subjects divided by quartiles of the 4 indices of visit-to-visit variability of HbA1c. Supplementary Figure 4: Kaplan-Meier curves for eGFR stage progression-free survival from G2 among subjects divided by quartiles of the 4 indices of visit-to-visit variability of HbA1c. Supplementary Figure 5: Kaplan-Meier curves for eGFR stage progression-free survival from G3 among subjects divided by quartiles of the 4 indices of visit-to-visit variability of HbA1c. Supplementary Figure 6: Kaplan-Meier curves for eGFR stage progression-free survival from G1 among PS-matched subjects from the Q1 and Q4 groups of each index of visit-to-visit variability of HbA1c. Supplementary Figure 7: seasonal changes affected HbA1c. Supplementary Table 1: value of the mean and quartiles of the 4 indices of visit-to-visit variability of HbA1c. Supplementary Table 2: baseline characteristics of all subjects divided by the quartiles of HbA1c-SD. Supplementary Table 3: baseline characteristics of all subjects divided by the quartiles of HbA1c-CV. Supplementary Table 4: baseline characteristics of all subjects divided by the quartiles of HbA1c-AUC. Supplementary Table 5: baseline characteristics of all subjects divided by the quartiles of HbA1c-HVS. Supplementary Table 6: association of the risk in eGFR stage progression from G1 with visit-to-visit variability of HbA1c, measured as 4 indices. The results were analyzed using a Cox proportional hazards model. Supplementary Table 7: association of the risk in eGFR stage progression from G2 and from G3 with visit-to-visit variability of HbA1c, measured as 4 indices. The results were analyzed using a Cox proportional hazards model. Supplementary Table 8: information of PS-matched subjects in the longitudinal analyses for the risk of microalbuminuria. Supplementary Table 9: information of PS-matched subjects in the longitudinal analyses for the risk of eGFR stage progression from G1.
