# Supplementary material for: Predictive Ability of Visit-to-Visit Variability of HbA1c Measurements for the Development of Diabetic Kidney Disease: A Retrospective Longitudinal Observational Study
Source: J Diabetes Res. 2022 Jan 22;2022:6934188. doi: 10.1155/2022/6934188 (PMC8800606; doi:10.1155/2022/6934188)
Supplement: Supplementary Materials — Supplementary Figure 1: study design to investigate the association between visit-to-visit variability of HbA1c and DKD risk. Supplementary Figure 2: calculation method of HbA1c-AUC. Supplementary Figure 3: Kaplan-Meier curves for eGFR stage progression-free survival from G1 among subjects divided by quartiles of the 4 indices of visit-to-visit variability of HbA1c. Supplementary Figure 4: Kaplan-Meier curves for eGFR stage progression-free survival from G2 among subjects divided by quartiles of the 4 indices of visit-to-visit variability of HbA1c. Supplementary Figure 5: Kaplan-Meier curves for eGFR stage progression-free survival from G3 among subjects divided by quartiles of the 4 indices of visit-to-visit variability of HbA1c. Supplementary Figure 6: Kaplan-Meier curves for eGFR stage progression-free survival from G1 among PS-matched subjects from the Q1 and Q4 groups of each index of visit-to-visit variability of HbA1c. Supplementary Figure 7: seasonal changes affected HbA1c. Supplementary Table 1: value of the mean and quartiles of the 4 indices of visit-to-visit variability of HbA1c. Supplementary Table 2: baseline characteristics of all subjects divided by the quartiles of HbA1c-SD. Supplementary Table 3: baseline characteristics of all subjects divided by the quartiles of HbA1c-CV. Supplementary Table 4: baseline characteristics of all subjects divided by the quartiles of HbA1c-AUC. Supplementary Table 5: baseline characteristics of all subjects divided by the quartiles of HbA1c-HVS. Supplementary Table 6: association of the risk in eGFR stage progression from G1 with visit-to-visit variability of HbA1c, measured as 4 indices. The results were analyzed using a Cox proportional hazards model. Supplementary Table 7: association of the risk in eGFR stage progression from G2 and from G3 with visit-to-visit variability of HbA1c, measured as 4 indices. The results were analyzed using a Cox proportional hazards model. Supplementary Table 8: information of PS-matc [file 6934188.f1.docx]

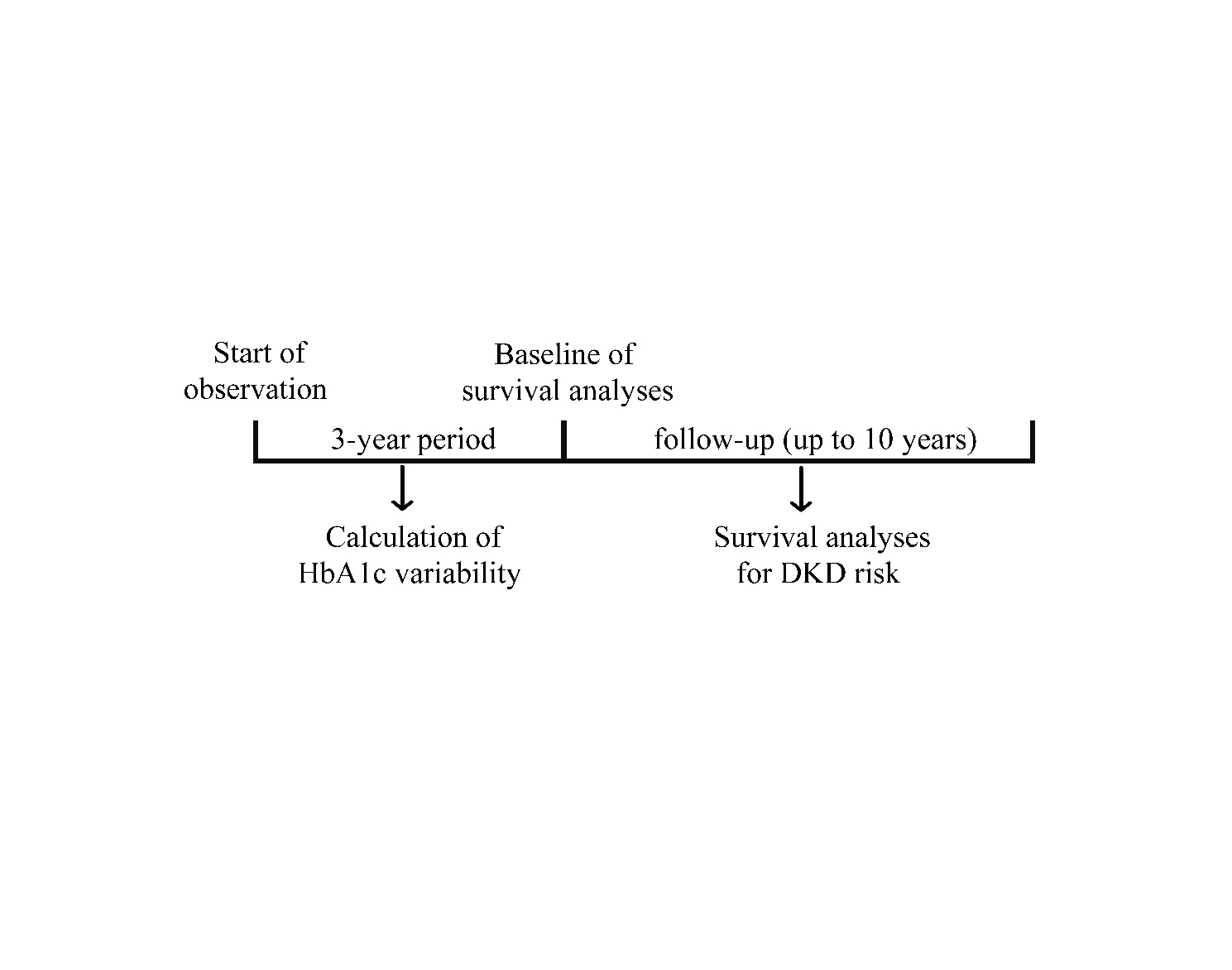


Supplementary Figure 1: Study design to investigate the association between visit-to-visit variability of HbA1c and DKD risk. DKD: diabetic kidney disease.


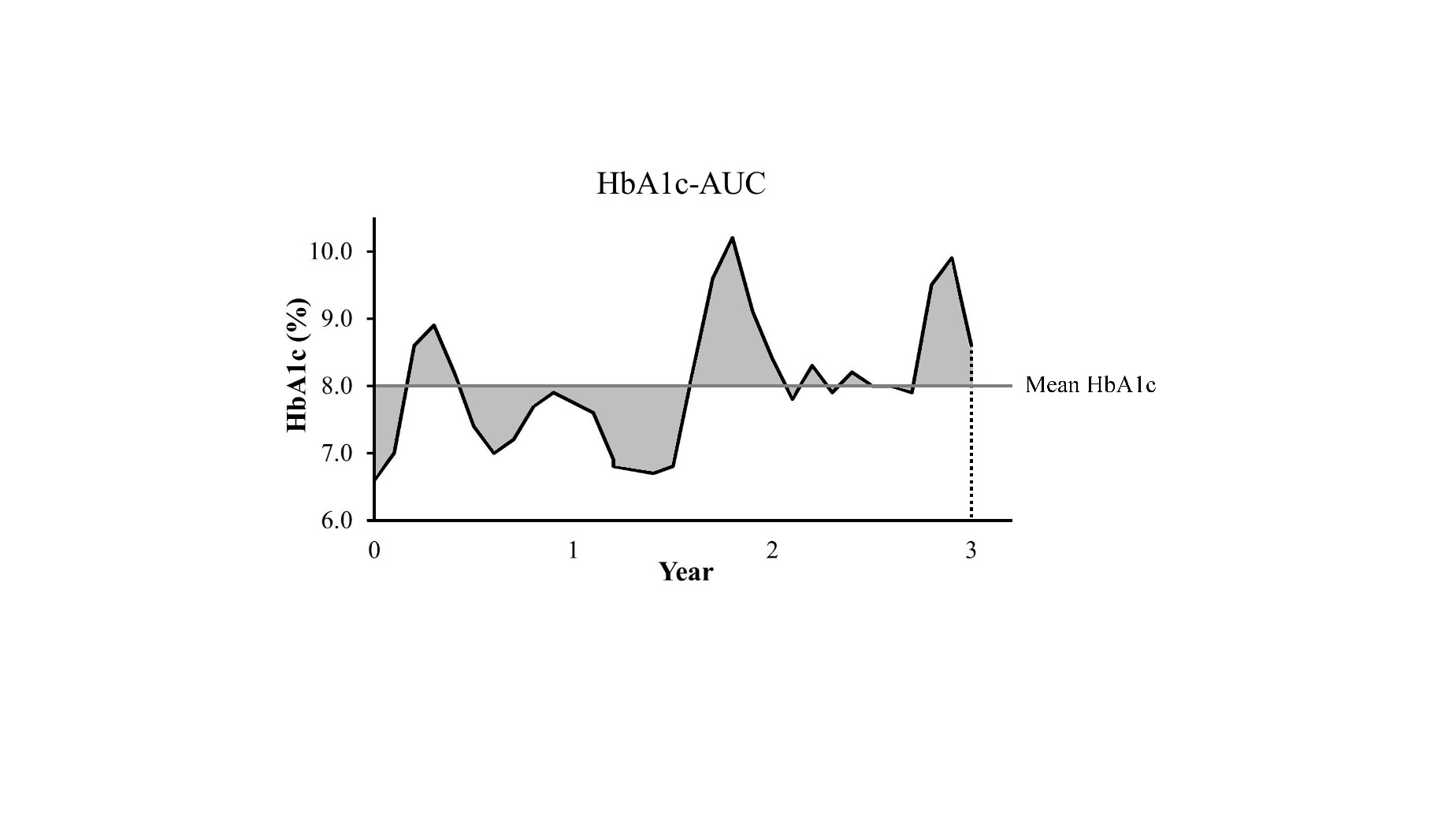


Supplementary Figure 2: Calculation method of HbA1c-AUC (Presented as the grey area).


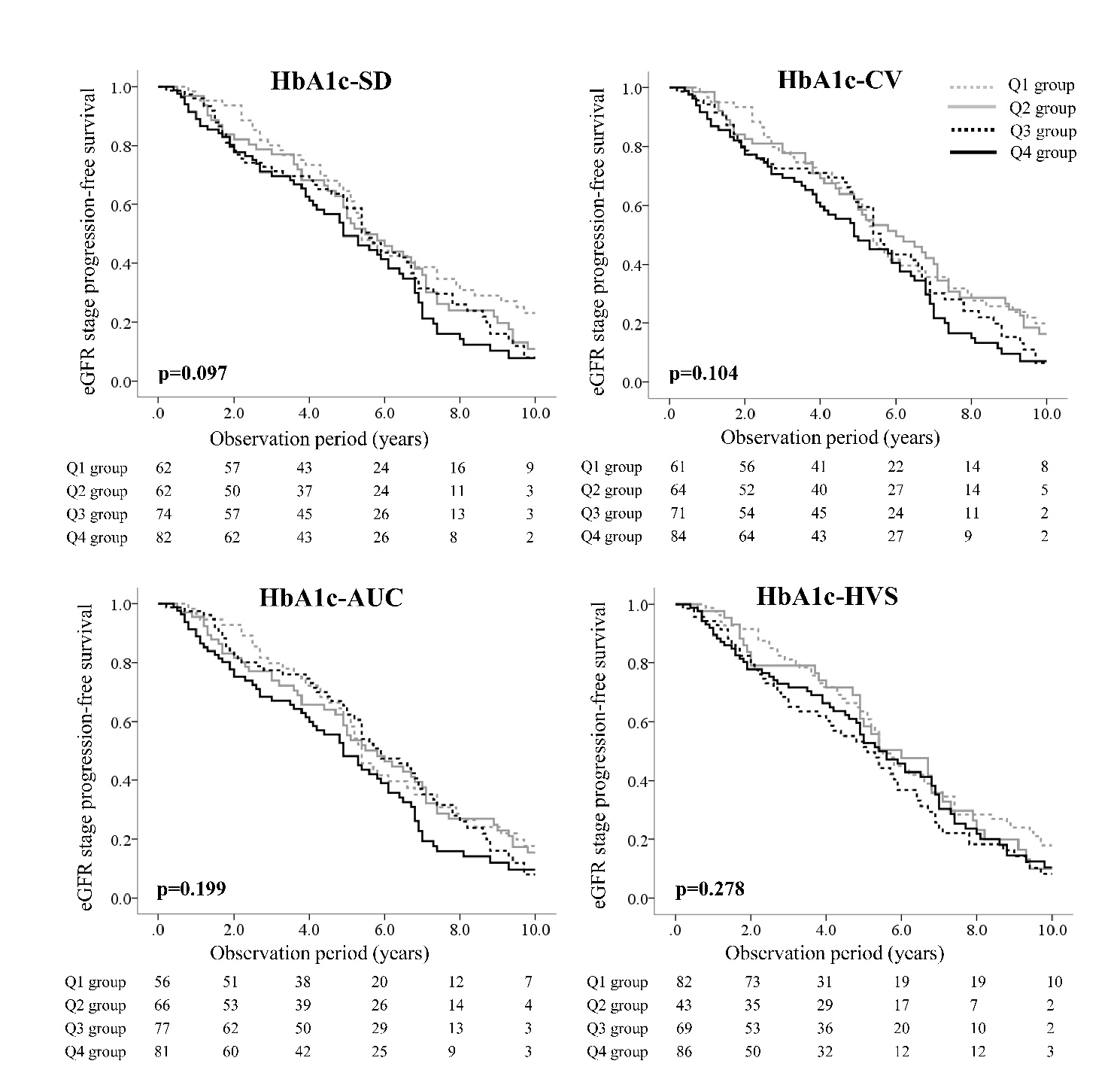


Supplementary Figure 3: Kaplan-Meier curves for eGFR stage progression-free survival from G1 among subjects divided by quartiles of the 4 indices of visit-to-visit variability of HbA1c. The quartiles of each index are represented by Q1, Q2, Q3 and Q4. The lower quartile is denoted as Q1, and the upper quartile is denoted as Q4. The comparison of the cumulative incidence among groups was carried out using a log rank test. eGFR: estimated glomerular filtration rate; HbA1c-SD: internal standard deviation of HbA1c; HbA1c-CV: coefficient of variation of HbA1c; HbA1c-AUC: area under the HbA1c curve; HbA1c-HVS: HbA1c change score.


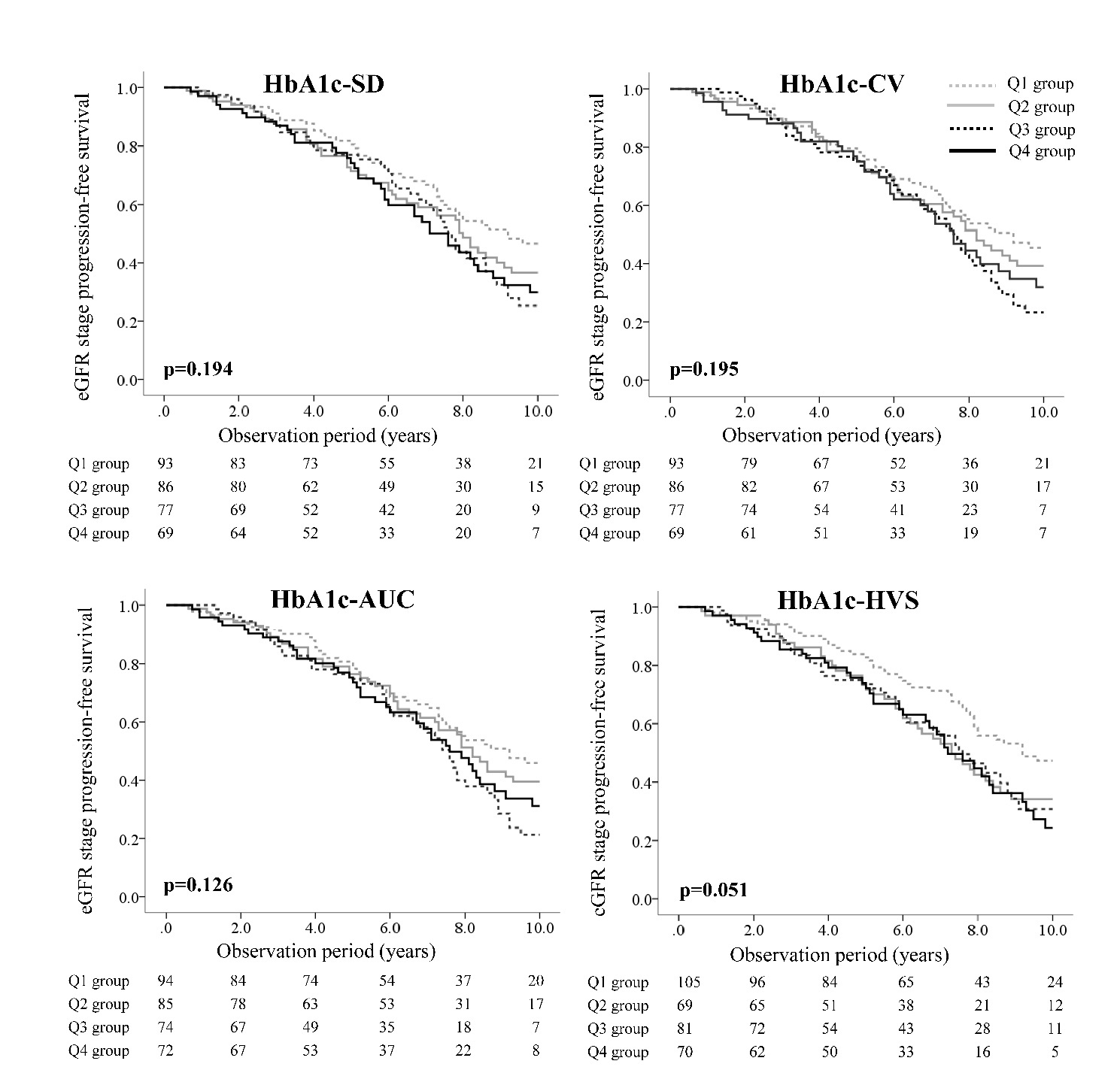


Supplementary Figure 4: Kaplan-Meier curves for eGFR stage progression-free survival from G2 among subjects divided by quartiles of the 4 indices of visit-to-visit variability of HbA1c. The quartiles of each index are represented by Q1, Q2, Q3 and Q4. The lower quartile is denoted as Q1, and the upper quartile is denoted as Q4. The comparison of the cumulative incidence among groups was carried out using a log rank test. eGFR: estimated glomerular filtration rate; HbA1c-SD: internal standard deviation of HbA1c; HbA1c-CV: coefficient of variation of HbA1c; HbA1c-AUC: area under the HbA1c curve; HbA1c-HVS: HbA1c change score.


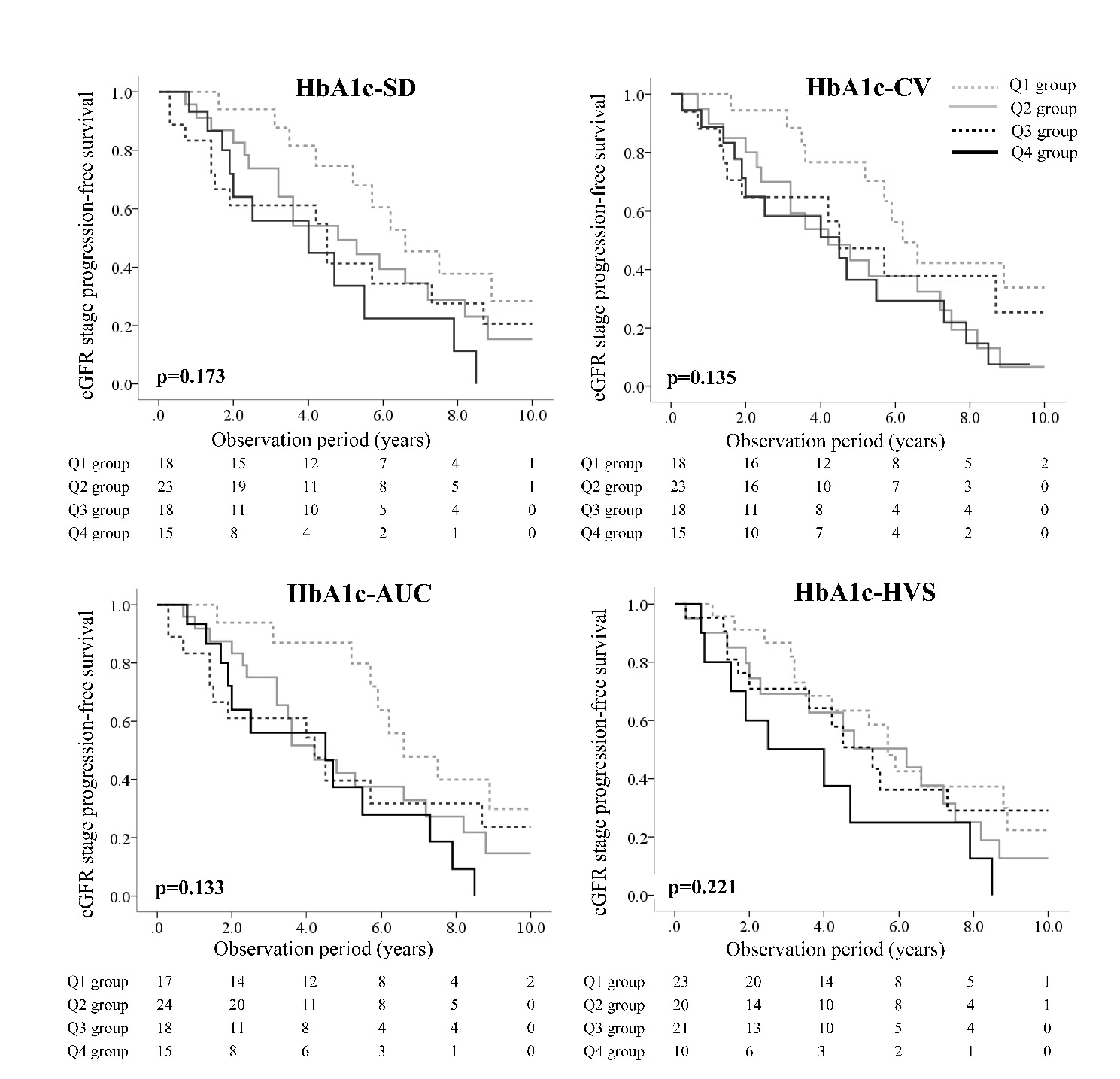


Supplementary Figure 5: Kaplan-Meier curves for eGFR stage progression-free survival from G3 among subjects divided by quartiles of the 4 indices of visit-to-visit variability of HbA1c. The quartiles of each index are represented by Q1, Q2, Q3 and Q4. The lower quartile is denoted as Q1, and the upper quartile is denoted as Q4. The comparison of the cumulative incidence among groups was carried out using a log rank test. eGFR: estimated glomerular filtration rate; HbA1c-SD: internal standard deviation of HbA1c; HbA1c-CV: coefficient of variation of HbA1c; HbA1c-AUC: area under the HbA1c curve; HbA1c-HVS: HbA1c change score.


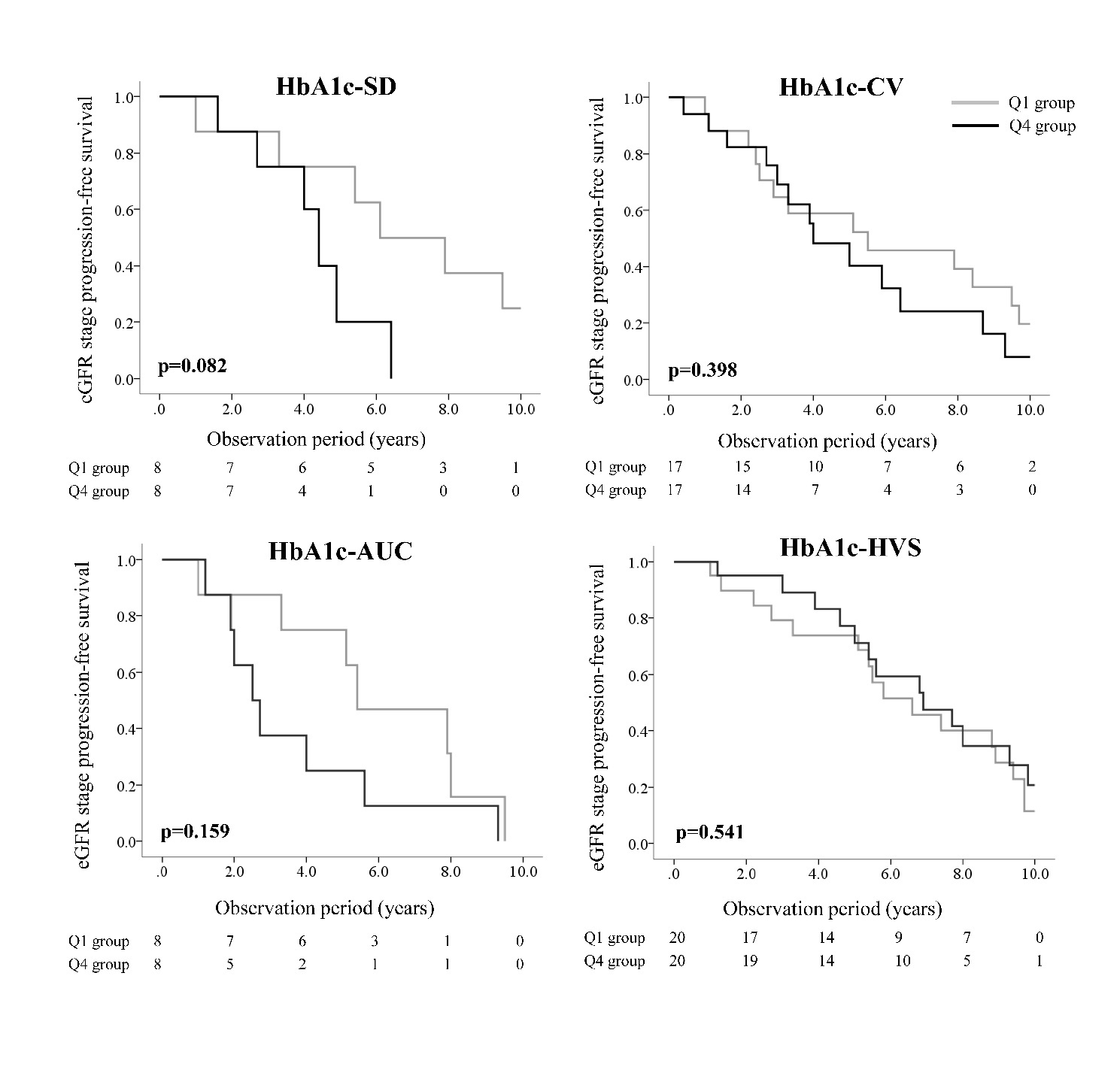


Supplementary Figure 6: Kaplan-Meier curves for eGFR stage progression-free survival from G1 among PS-matched subjects from the Q1 and Q4 groups of each index of visit-to-visit variability of HbA1c. The lower quartile is denoted as Q1, and the upper quartile is denoted as Q4. The comparison of the cumulative incidence among groups was carried out using a log rank test. eGFR: estimated glomerular filtration rate; HbA1c-SD: internal standard deviation of HbA1c; HbA1c-CV: coefficient of variation of HbA1c; HbA1c-AUC: area under the HbA1c curve; HbA1c-HVS: HbA1c change score.


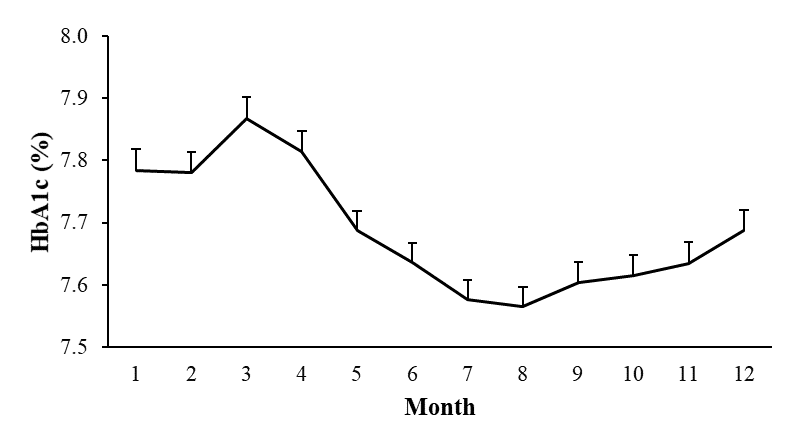


Supplementary Figure 7: Seasonal changes affected HbA1c. The peak and tough of HbA1c values appeared in March and August, respectively. The error bars represent the standard error of HbA1c measurements at each month (average numbers of HbA1c values at each month: 1602 values).

Supplementary Table 1: Value of the mean and quartiles of the 4 indices of visit-to-visit variability of HbA1c.

| Index | Mean | Q1 | Q2 | Q3 | Q4 |
| --- | --- | --- | --- | --- | --- |
| HbA1c-SD | 0.59 | [0.05, 0.27] | (0.27, 0.47] | (0.47, 0.76] | (0.76, 3.01] |
| HbA1c-CV | 7.38 | [0.84, 3.82] | (3.82, 6.06] | (6.06, 9.63] | (9.63, 33.7] |
| HbA1c-AUC | 1.41 | [0.08, 0.62] | (0.62, 1.13] | (1.13, 1.83] | (1.83, 9.86] |
| HbA1c-HVS | 15.69 | [0.00, 0.00] | (0.00, 9.68] | (9.68, 25.0] | (25.0, 85.7] |

The quartiles of each index are represented by Q1, Q2, Q3 and Q4. The lower quartile is denoted as Q1, and the upper quartile is denoted as Q4. HbA1c-SD: internal standard deviation of HbA1c; HbA1c-CV: coefficient of variation of HbA1c; HbA1c-AUC: area under the HbA1c curve; HbA1c-HVS: HbA1c change score.

Supplementary Table 2: Baseline characteristics of all subjects divided by the quartiles of HbA1c-SD.

|  | HbA1c-SD | | | | |
| --- | --- | --- | --- | --- | --- |
|  | Q1 | Q2 | Q3 | Q4 | p value |
|  | (n = 180) | (n = 172) | (n = 174) | (n = 173) |  |
| Male/Female | 121 / 59 | 131 / 41 | 111 / 63 | 114 / 59 | 0.07 |
| Age (years) | 61.0 ± 9.0 | 60.3 ± 10.5 | 59.4 ± 10.7 | 55.7 ± 10.7 | <0.01 |
| Duration of diabetes (years) | 10.0 ± 7.4 | 14.0 ± 8.9 | 12.2 ± 7.4 | 11.7 ± 6.7 | <0.01 |
| HbA1c (%) | 6.8 ± 0.8 | 7.4 ± 0.9 | 8.0 ± 1.2 | 8.8 ± 1.9 | <0.01 |
| BMI (kg/m^2^) | 23.1 ± 3.3 | 24.0 ± 3.5 | 25.1 ± 3.8 | 26.2 ± 4.9 | <0.01 |
| SBP (mmHg) | 130.6 ± 17.2 | 136.2 ± 17.1 | 138.4 ± 18.0 | 137.3 ± 18.2 | <0.01 |
| DBP (mmHg) | 79.7 ± 10.3 | 81.2 ± 9.9 | 84.0 ± 11.2 | 83.6 ± 10.9 | <0.01 |
| HDL cholesterol (mmol/L) | 1.5 ± 0.4 | 1.5 ± 0.5 | 1.4 ± 0.4 | 1.4 ± 0.5 | 0.09 |
| LDL cholesterol (mmol/L) | 3.1 ± 0.8 | 3.1 ± 0.8 | 3.2 ± 0.9 | 3.4 ± 1.0 | 0.01 |
| Triglycerides (mmol/L) | 1.3 ± 1.0 | 1.5 ± 1.1 | 1.8 ± 1.5 | 2.0 ± 1.7 | <0.01 |
| **3-year follow-up after the start of observation** | | | | | |
| Mean HbA1c (%) | 6.6 ± 0.6 | 7.4 ± 0.8 | 8.0 ± 0.9 | 8.7 ± 1.0 | <0.01 |
| Number of HbA1c measurements (times) | 25.3 ± 10.8 | 29.1 ± 8.7 | 28.0 ± 9.3 | 27.7 ± 9.0 | <0.01 |

Data are shown as the number or the mean ± SD. The quartiles of HbA1c-SD are represented by Q1, Q2, Q3 and Q4. The lower quartile is denoted as Q1, and the upper quartile is denoted as Q4. HbA1c-SD: internal standard deviation of HbA1c; BMI: body mass index; SBP: systolic blood pressure; DBP: diastolic blood pressure; HDL: high-density lipoprotein; LDL: low-density lipoprotein; SD: standard deviation.

Supplementary Table 3: Baseline characteristics of all subjects divided by the quartiles of HbA1c-CV.

|  | HbA1c-CV | | | | |
| --- | --- | --- | --- | --- | --- |
|  | Q1 | Q2 | Q3 | Q4 | p value |
|  | (n = 176) | (n = 174) | (n = 175) | (n = 174) |  |
| Male/Female | 115 / 61 | 138 / 36 | 110 / 65 | 114 / 60 | <0.01 |
| Age (years) | 61.0 ± 9.0 | 60.3 ± 10.2 | 58.7 ±11.1 | 56.5 ± 10.7 | <0.01 |
| Duration of diabetes (years) | 10.6 ± 7.8 | 13.5 ± 8.9 | 12.3 ± 7.2 | 11.6 ± 6.8 | <0.01 |
| HbA1c (%) | 6.9 ± 0.9 | 7.5 ± 0.9 | 8.0 ± 1.3 | 8.6 ± 1.9 | <0.01 |
| BMI (kg/m^2^) | 23.1 ± 3.3 | 23.9 ± 3.9 | 25.0 ± 3.6 | 26.2 ± 4.9 | <0.01 |
| SBP (mmHg) | 131.0 ± 17.9 | 135.1 ± 16.5 | 138.2 ± 17.4 | 138.0 ± 18.8 | <0.01 |
| DBP (mmHg) | 79.7 ± 10.5 | 81.2 ± 9.7 | 83.6 ± 10.5 | 84.0 ± 11.4 | 0.09 |
| HDL cholesterol (mmol/L) | 1.5 ± 0.4 | 1.4 ± 0.4 | 1.4 ± 0.5 | 1.4 ± 0.4 | 0.05 |
| LDL cholesterol (mmol/L) | 3.1 ± 0.8 | 3.1 ± 0.8 | 3.2 ± 0.9 | 3.4 ± 1.0 | <0.01 |
| Triglycerides (mmol/L) | 1.3 ± 1.0 | 1.5 ±1.1 | 1.8 ± 1.6 | 1.9 ± 1.6 | <0.01 |
| **3-year follow-up after the start of observation** | | | | | |
| Mean HbA1c (%) | 6.7 ± 0.8 | 7.5 ± 0.9 | 8.0 ± 1.0 | 8.5 ± 1.1 | <0.01 |
| Number of HbA1c measurements (times) | 25.4 ± 10.8 | 28.9 ± 9.0 | 28.0 ± 9.2 | 27.8 ± 8.9 | <0.01 |

Data are shown as the number or the mean ± SD. The quartiles of HbA1c-CV are represented by Q1, Q2, Q3 and Q4. The lower quartile is denoted as Q1, and the upper quartile is denoted as Q4. HbA1c-CV: coefficient of variation of HbA1c; BMI: body mass index; SBP: systolic blood pressure; DBP: diastolic blood pressure; HDL: high-density lipoprotein; LDL: low-density lipoprotein; SD: standard deviation.

Supplementary Table 4: Baseline characteristics of all subjects divided by the quartiles of HbA1c-AUC.

|  | HbA1c-AUC | | | | |
| --- | --- | --- | --- | --- | --- |
|  | Q1 | Q2 | Q3 | Q4 | p value |
|  | (n = 175) | (n = 177) | (n = 173) | (n = 174) |  |
| Male/Female | 117 / 58 | 138 / 39 | 107 / 72 | 121 / 53 | <0.01 |
| Age (years) | 61.2 ± 9.1 | 59.5 ± 10.2 | 60.2 ± 10.8 | 55.5 ± 10.6 | <0.01 |
| Duration of diabetes (years) | 10.8 ± 8.3 | 13.1 ± 8.3 | 12.8 ± 7.6 | 11.1 ± 6.6 | <0.01 |
| HbA1c (%) | 6.7 ± 0.7 | 7.5 ± 0.9 | 7.9 ± 1.2 | 8.9 ± 1.9 | <0.01 |
| BMI (kg/m^2^) | 23 ± 3.3 | 24.1 ± 3.5 | 24.9 ± 3.9 | 26.2 ± 4.9 | <0.01 |
| SBP (mmHg) | 130.6 ± 17.5 | 137.3 ± 17.3 | 136.7 ± 17.1 | 137.6 ± 18.7 | <0.01 |
| DBP (mmHg) | 79.6 ± 10.3 | 82.0 ± 10.2 | 83.0 ± 10.5 | 83.9 ± 11.2 | <0.01 |
| HDL cholesterol (mmol/L) | 1.5 ± 0.5 | 1.5 ± 0.4 | 1.4 ± 0.5 | 1.4 ± 0.4 | 0.04 |
| LDL cholesterol (mmol/L) | 3.1 ± 0.8 | 3.1 ± 0.8 | 3.2 ± 0.8 | 3.4 ± 1.0 | 0.01 |
| Triglycerides (mmol/L) | 1.3 ± 1.0 | 1.5 ± 1.1 | 1.7 ± 1.6 | 2.0 ± 1.6 | <0.01 |
| **3-year follow-up after the start of observation** | | | | | |
| Mean HbA1c (%) | 6.6 ± 0.7 | 7.4 ± 0.8 | 8.0 ± 0.9 | 8.7 ± 1.1 | <0.01 |
| Number of HbA1c measurements (times) | 25.8 ± 10.4 | 28.7 ± 9.1 | 28.6 ± 9.0 | 26.9 ± 9.5 | 0.01 |

Data are shown as the number or the mean ± SD. The quartiles of HbA1c-AUC are represented by Q1, Q2, Q3 and Q4. The lower quartile is denoted as Q1, and the upper quartile is denoted as Q4. HbA1c-AUC: area under the HbA1c curve; BMI: body mass index; SBP: systolic blood pressure; DBP: diastolic blood pressure; HDL: high-density lipoprotein; LDL: low-density lipoprotein; SD: standard deviation.

Supplementary Table 5: Baseline characteristics of all subjects divided by the quartiles of HbA1c-HVS.

|  | HbA1c-HVS | | | | |
| --- | --- | --- | --- | --- | --- |
|  | Q1 | Q2 | Q3 | Q4 | p value |
|  | (n = 217) | (n = 133) | (n = 179) | (n = 170) |  |
| Male/Female | 148 / 69 | 96 / 37 | 107 / 72 | 126 / 44 | 0.02 |
| Age (years) | 59.8 ± 9.6 | 62.4 ± 9.8 | 59.3 ± 10.1 | 55.4 ± 11.1 | <0.01 |
| Duration of diabetes (years) | 10.6 ± 7.9 | 14.1 ± 8.8 | 12.6 ± 7.4 | 11.5 ± 6.8 | <0.01 |
| HbA1c (%) | 6.9 ± 0.8 | 7.5 ± 0.8 | 7.9 ± 1.3 | 8.9 ± 1.8 | <0.01 |
| BMI (kg/m^2^) | 23.4 ± 3.6 | 24.2 ± 3.5 | 24.8 ± 3.6 | 26.1 ± 5.0 | <0.01 |
| SBP (mmHg) | 131.5 ± 16.8 | 136.5 ± 17.9 | 136.0 ± 15.9 | 139.6 ± 19.9 | <0.01 |
| DBP (mmHg) | 79.9 ± 10.1 | 81.6 ± 10.1 | 81.9 ± 10.4 | 85.6 ± 11.3 | <0.01 |
| HDL cholesterol (mmol/L) | 1.5 ± 0.4 | 1.4 ± 0.5 | 1.4 ± 0.4 | 1.4 ± 0.4 | 0.09 |
| LDL cholesterol (mmol/L) | 3.2 ± 0.8 | 3.1 ± 0.8 | 3.2 ± 0.9 | 3.2 ± 1.0 | 0.47 |
| Triglycerides (mmol/L) | 1.4 ± 1.2 | 1.5 ± 1.0 | 1.7 ± 1.3 | 2.0 ± 1.8 | <0.01 |
| **3-year follow-up after the start of observation** | | | | | |
| Mean HbA1c (%) | 6.7 ± 0.7 | 7.4 ± 0.7 | 7.9 ± 0.9 | 8.8 ± 1.1 | <0.01 |
| Number of HbA1c measurements (times) | 26.9 ± 10.3 | 32.2 ± 5.7 | 28.3 ± 9.3 | 23.8 ± 9.7 | <0.01 |

Data are shown as the number or the mean ± SD. The quartiles of HbA1c- HVS are represented by Q1, Q2, Q3 and Q4. The lower quartile is denoted as Q1, and the upper quartile is denoted as Q4. HbA1c-HVS: HbA1c change score; BMI: body mass index; SBP: systolic blood pressure; DBP: diastolic blood pressure; HDL: high-density lipoprotein; LDL: low-density lipoprotein; SD: standard deviation.

Supplementary Table 6: Association of the risk in eGFR stage progression from G1 with visit-to-visit variability of HbA1c, measured as 4 indices. The results were analysed using a Cox proportional hazards model.

| Index | HR [95%CI] ^a^ | p value ^b^ | p value ^c^ | PS-matched subjects | |
| --- | --- | --- | --- | --- | --- |
|  |  |  |  | HR [95%CI] | p value ^b^ |
| HbA1c-SD |  |  |  |  |  |
| Q1 | 1 | - | - | 1 | - |
| Q2 | 1.30 (0.83-2.03) | 0.254 | 0.289 |  |  |
| Q3 | 1.58 (0.97-2.56) | 0.065 | 0.075 |  |  |
| Q4 | 1.89 (1.11-3.24) | 0.020 | 0.025 | 3.13 (0.82-11.93) | 0.094 |
| HbA1c-CV |  |  |  |  |  |
| Q1 | 1 | - | - | 1 | - |
| Q2 | 0.97 (0.63-1.51) | 0.907 | 0.907 |  |  |
| Q3 | 1.40 (0.88-2.22) | 0.159 | 0.184 |  |  |
| Q4 | 1.67 (1.03-2.71) | 0.039 | 0.048 | 1.40 (0.64-3.07) | 0.401 |
| HbA1c-AUC |  |  |  |  |  |
| Q1 | 1 | - | - | 1 | - |
| Q2 | 1.05 (0.67-1.65) | 0.830 | 0.817 |  |  |
| Q3 | 1.08 (0.67-1.74) | 0.749 | 0.770 |  |  |
| Q4 | 1.50 (0.90-2.51) | 0.123 | 0.145 | 2.14 (0.73-6.28) | 0.168 |
| HbA1c-HVS |  |  |  |  |  |
| Q1 | 1 | - | - | 1 | - |
| Q2 | 0.97 (0.62-1.52) | 0.910 | 0.910 |  |  |
| Q3 | 1.21 (0.80-1.85) | 0.365 | 0.409 |  |  |
| Q4 | 1.04 (0.67-1.61) | 0.859 | 0.847 | 0.80 (0.38-1.66) | 0.544 |

The quartiles of each index are represented by Q1, Q2, Q3 and Q4. The lower quartile is denoted as Q1, and the upper quartile is denoted as Q4. ^a^ Adjusted by age, sex, mean HbA1c over the 3-year period, duration of diabetes, SBP, LDL-c, eGFR, presence of ischemic heart disease and heart failure at baseline. ^b^ Analysed using a Cox proportional hazards model. ^c^ Analysed using a bootstrap analysis based on 1,000 replicated data sets. eGFR: estimated glomerular filtration rate; HbA1c-SD: internal standard deviation of HbA1c; HbA1c-CV: coefficient of variation of HbA1c; HbA1c-AUC: area under the HbA1c curve; HbA1c-HVS: HbA1c change score; HR: hazard ratio; CI: confidence interval.

Supplementary Table 7: Association of the risk in eGFR stage progression from G2 and from G3 with visit-to-visit variability of HbA1c, measured as 4 indices. The results were analysed using a Cox proportional hazards model.

| Index | eGFR stage progression from G2 | |  | eGFR stage progression from G3 | |
| --- | --- | --- | --- | --- | --- |
|  | HR [95%CI] ^a^ | p value |  | HR [95%CI] ^a^ | p value |
| HbA1c-SD |  |  |  |  |  |
| Q1 | 1 | - |  | 1 | - |
| Q2 | 1.20 (0.76-1.90) | 0.434 |  | 1.30 (0.48-3.49) | 0.606 |
| Q3 | 1.05 (0.63-1.76) | 0.854 |  | 0.74 (0.26-2.15) | 0.585 |
| Q4 | 1.55 (0.86-2.77) | 0.142 |  | 1.38 (0.38-5.06) | 0.623 |
| HbA1c-CV |  |  |  |  |  |
| Q1 | 1 | - |  | 1 | - |
| Q2 | 1.17 (0.73-1.87) | 0.516 |  | 2.36 (0.98-5.71) | 0.056 |
| Q3 | 1.08 (0.66-1.75) | 0.756 |  | 0.74 (0.26-2.10) | 0.573 |
| Q4 | 1.41 (0.80-2.47) | 0.236 |  | 1.90 (0.65-5.53) | 0.241 |
| HbA1c-AUC |  |  |  |  |  |
| Q1 | 1 | - |  | 1 | - |
| Q2 | 1.08 (0.68-1.70) | 0.752 |  | 1.71 (0.65-4.49) | 0.273 |
| Q3 | 1.05 (0.63-1.78) | 0.842 |  | 0.85 (0.29-2.51) | 0.775 |
| Q4 | 1.41 (0.79-2.52) | 0.247 |  | 1.67 (0.51-5.46) | 0.400 |
| HbA1c-HVS |  |  |  |  |  |
| Q1 | 1 | - |  | 1 | - |
| Q2 | 1.51 (0.95-2.40) | 0.083 |  | 1.41 (0.56-3.52) | 0.463 |
| Q3 | 1.49 (0.94-2.38) | 0.092 |  | 0.54 (0.20-1.47) | 0.229 |
| Q4 | 1.55 (0.84-2.86) | 0.160 |  | 1.36 (0.39-4.82) | 0.629 |

The quartiles of each index are represented by Q1, Q2, Q3 and Q4. The lower quartile is denoted as Q1, and the upper quartile is denoted as Q4. ^a^ Adjusted by age, sex, mean HbA1c over the 3-year period, duration of diabetes, SBP, LDL-C, eGFR, presence of ischemic heart disease and heart failure at baseline. eGFR: estimated glomerular filtration rate; HbA1c-SD: internal standard deviation of HbA1c; HbA1c-CV: coefficient of variation of HbA1c; HbA1c-AUC: area under the HbA1c curve; HbA1c-HVS: HbA1c change score; HR: hazard ratio; CI: confidence interval; SBP systolic blood pressure; LDL-C: low-density lipoprotein cholesterol.

Supplementary Table 8: Information of PS-matched subjects in the longitudinal analyses for the risk of microalbuminuria.

|  | HbA1c-SD | | p value | | | HbA1c-CV | | | | | p value | |
| --- | --- | --- | --- | --- | --- | --- | --- | --- | --- | --- | --- | --- |
|  | Q1 | Q4 |  |  |  | Q1 | | | Q4 | |  |  |
| N | 14 | 14 | | | - | 14 | | | 14 | | - | |
| Males/females | 9 / 5 | 8 / 6 | | | - | 9 / 5 | | | 8 / 6 | | - | |
| Age (years) | 60.1 ± 8.8 | 59.3 ± 7.2 | | | 0.79 | 61.2 ± 6.5 | | | 60.8 ± 9.1 | | 0.90 | |
| Duration of diabetes (years) | 11.6 ± 7.9 | 11.7 ± 7.9 | | | 0.99 | 11.9 ± 7.6 | | | 10.6 ± 7.2 | | 0.66 | |
| HbA1c (%) | 7.5 ± 0.6 | 7.5 ± 1.3 | | | 0.94 | 7.3 ± 0.8 | | | 7.7 ± 1.3 | | 0.34 | |
| Mean HbA1c (%) | 7.3 ± 0.6 | 7.3 ± 0.6 | | | 0.90 | 7.3 ± 0.7 | | | 7.3 ± 0.7 | | 0.96 | |
| SBP (mmHg) | 129.8 ± 21.2 | 133.9 ± 14.5 | | | 0.55 | 138.5 ± 23.1 | | | 137.1 ± 18.0 | | 0.86 | |
|  | HbA1c-AUC | | | p value | | | HbA1c-HVS | | | p value | |  |
|  | Q1 | Q4 | |  |  |  | Q1 | Q4 | |  |  |  |
| N | 15 | 15 | | - | | | 20 | 20 | | - | |  |
| Males/females | 11 / 4 | 8 / 7 | | - | | | 13 / 7 | 14 / 6 | | - | |  |
| Age (years) | 61.3 ± 8.0 | 60.2 ± 9.8 | | 0.74 | | | 55.7 ± 10.5 | 58.4 ± 11.7 | | 0.45 | |  |
| Duration of diabetes (years) | 11.7 ± 8.0 | 10.4 ± 6.7 | | 0.62 | | | 11.3 ± 6.6 | 11.6 ± 7.7 | | 0.90 | |  |
| HbA1c (%) | 7.2 ± 0.7 | 7.5 ± 1.8 | | 0.58 | | | 7.9 ± 1.1 | 7.8 ± 1.6 | | 0.84 | |  |
| Mean HbA1c (%) | 7.3 ± 0.6 | 7.3 ± 0.7 | | 0.91 | | | 7.6 ± 0.6 | 7.6 ± 0.8 | | 0.98 | |  |
| SBP (mmHg) | 137.7 ± 16.4 | 139.8 ± 16.9 | | 0.73 | | | 137.7 ± 13.4 | 140.3 ± 19.1 | | 0.62 | |  |

Data are shown as the number or the mean ± SD. The lower quartile is denoted as Q1, and the upper quartile is denoted as Q4. PS: propensity score; HbA1c-SD: internal standard deviation of HbA1c; HbA1c-CV: coefficient of variation of HbA1c; HbA1c-AUC: area under the HbA1c curve; HbA1c-HVS: HbA1c change score; SBP: systolic blood pressure; SD: standard deviation.

Supplementary Table 9. Information of PS-matched subjects in the longitudinal analyses for the risk of eGFR stage progression from G1.

|  | HbA1c-SD | | p value | HbA1c-CV | | p value |
| --- | --- | --- | --- | --- | --- | --- |
|  | Q1 | Q4 |  | Q1 | Q4 |  |
| N | 8 | 8 | - | 17 | 17 | - |
| Males/females | 5/3 | 3/5 | - | 7/10 | 10/7 | - |
| Age (years) | 60.2 ± 9.7 | 58.2 ± 8.1 | 0.66 | 57.5 ± 10.1 | 55.4 ± 8.7 | 0.51 |
| Duration of diabetes (years) | 12.1 ± 7.8 | 7.0 ± 3.1 | 0.11 | 9.8 ± 5.7 | 10.9 ± 6.7 | 0.60 |
| HbA1c (%) | 7.5 ± 0.7 | 8.3 ± 2.2 | 0.31 | 7.9 ± 1.3 | 8.1 ± 2.0 | 0.63 |
| Mean HbA1c (%) | 7.4 ± 0.6 | 7.4 ± 0.9 | 0.90 | 7.5 ± 0.7 | 7.5 ± 0.8 | 0.89 |
| SBP (mmHg) | 130.3±27.2 | 136.0 ± 16.1 | 0.62 | 133.6 ± 21.7 | 139.2 ± 22.6 | 0.47 |
|  | HbA1c-AUC | | p value | HbA1c-HVS | | p value |
|  | Q1 | Q4 |  | Q1 | Q4 |  |
| N | 8 | 8 | - | 20 | 20 | - |
| Males/females | 3/5 | 3/5 | - | 12/8 | 12/8 | - |
| Age (years) | 57.0 ± 9.7 | 59.4 ± 7.9 | 0.59 | 52.6 ± 9.9 | 56.6 ± 10.4 | 0.22 |
| Duration of diabetes (years) | 10.9 ± 4.8 | 9.0 ± 6.3 | 0.53 | 9.8 ± 5.5 | 10.1 ± 6.1 | 0.86 |
| HbA1c (%) | 7.5 ± 0.6 | 7.3 ± 1.4 | 0.66 | 7.8 ± 1.3 | 7.9 ± 1.4 | 0.88 |
| Mean HbA1c (%) | 7.4 ± 0.6 | 7.4 ± 0.8 | 0.89 | 7.7 ± 0.7 | 7.7 ± 0.8 | 0.87 |
| SBP (mmHg) | 142.0 ± 26.6 | 133.4 ± 21.8 | 0.49 | 135.3 ± 16.0 | 134.0 ± 19.5 | 0.82 |

Data are shown as the number or the mean ± SD. The lower quartile is denoted as Q1, and the upper quartile is denoted as Q4. PS: propensity score; eGFR: estimated glomerular filtration rate; HbA1c-SD: internal standard deviation of HbA1c; HbA1c-CV: coefficient of variation of HbA1c; HbA1c-AUC: area under the HbA1c curve; HbA1c-HVS: HbA1c change score; SBP: systolic blood pressure; SD: standard deviation.
